# Effects of chronic hypercapnia on ammonium transport in the mouse kidney

**DOI:** 10.14814/phy2.14221

**Published:** 2019-08-27

**Authors:** Solange Abdulnour‐Nakhoul, Kathleen Hering‐Smith, L. Lee Hamm, Nazih L. Nakhoul

**Affiliations:** ^1^ Section of Nephrology, Departments of Medicine and Physiology Tulane University School of Medicine New Orleans Louisiana

**Keywords:** Rh glycoproteins, NH_3_/NH_4_^+^, hypercapnia, respiratory acidosis, acid–base, pH homeostasis

## Abstract

Hypercapnia and subsequent respiratory acidosis are serious complications in many patients with respiratory disorders. The acute response to hypercapnia is buffering of H^+^ by hemoglobin and cellular proteins but this effect is limited. The chronic response is renal compensation that increases HCO_3_^−^ reabsorption, and stimulates urinary excretion of titratable acids (TA) and NH_4_^+^. However, the main effective pathway is the excretion of NH_4_^+^ in the collecting duct. Our hypothesis is that, the renal NH_3_/NH_4_^+^ transporters, Rhbg and Rhcg, in the collecting duct mediate this response. The effect of hypercapnia on these transporters is unknown. We conducted in vivo experiments on mice subjected to chronic hypercapnia. One group breathed 8% CO_2_ and the other breathed normal air as control (0.04% CO_2_). After 3 days, the mice were euthanized and kidneys, blood, and urine samples were collected. We used immunohistochemistry and Western blot analysis to determine the effects of high CO_2_ on localization and expression of the Rh proteins, carbonic anhydrase IV, and pendrin. In hypercapnic animals, there was a significant increase in urinary NH_4_^+^ excretion but no change in TA. Western blot analysis showed a significant increase in cortical expression of Rhbg (43%) but not of Rhcg. Expression of CA‐IV was increased but pendrin was reduced. These data suggest that hypercapnia leads to compensatory upregulation of Rhbg that contributes to excretion of NH_3_/NH_4_^+^ in the kidney. These studies are the first to show a link among hypercapnia, NH_4_^+^ excretion, and Rh expression.

## Introduction

In the clinical setting, hypercapnia and subsequent respiratory acidosis are common and serious complications observed in patients with respiratory disorders such as chronic obstructive pulmonary disease (COPD) and acute respiratory distress syndrome (Bruno and Valenti, 2012). Acutely, hypercapnia causes a rapid increase in H^+^ concentration due to hydration of CO_2_, catalyzed by carbonic anhydrase (CA), and dissociation of H_2_CO_3_ (CO_2_ + H_2_O → H_2_CO_3_ → H^+^ + HCO_3_^−^). The acute response to hypercapnia is buffering of H^+^ by hemoglobin and other cellular proteins but this effect is limited. The chronic response to hypercapnia is renal compensation that increases HCO_3_^−^ reabsorption and stimulates urinary acid excretion leading to recovery of systemic pH.

The adaptive response of the kidney to acidosis in general (usually complete in 1–3 days) is mainly reflected in changes in renal ammonia metabolism that increase the capacity of the kidney to produce ammonia, alpha‐ketoglutarate (*α*‐KG), and glucose (Tannen, 1978, 1983). Ammoniagenesis occurs by deamination of glutamine to glutamic acid and then to *α*‐KG resulting in the production of two molecules of ammonia (NH_3_) for each molecule of glutamine. Further metabolism of *α*‐KG leads to the production of glucose (Tannen, 1978; Weiner and Verlander, 2013). The ammoniagenic response occurs mainly in the proximal tubule leading to increased HCO_3_^−^ reabsorption and an increase in urinary excretion of titratable acids (TA) and ammonium (NH_4_^+^).

The renal response to hypercapnia is complicated by temporal changes that differ from the observed changes caused by metabolic increase in H^+^. Acute acidosis, metabolic and respiratory, decreases cortical concentration of *α*‐KG (Trivedi and Tannen, 1986). Similarly, acute, but not chronic, respiratory acidosis was reported to increase renal ammonia and gluconeogenesis in rats (Rodriguez‐Nichols et al., 1984). These effects are thought to be caused by a decrease in intracellular pH (pH_i_) of the proximal tubule (Trivedi and Tannen, 1986). In the chronic phase (1–3 days), low pH_i_ is maintained in metabolic acidosis but is recovered, at least partially, in respiratory acidosis and there are no apparent further effects on production of *α*‐KG. However, other studies (Kamm et al., 1967) reported that chronic respiratory acidosis stimulated glucose production from *α*‐KG and resulted in persistent increase in NH_4_^+^ and net acid excretion (NAE) (Polak et al., 1961; De Sousa et al., 1974).

Increased renal production of NH_3_/NH_4_^+^ is accompanied by adaptive changes in tubular transport for proper compensation during prolonged respiratory acidosis. In the proximal tubule, chronic hypercapnia stimulated HCO_3_^−^ reabsorption to a level higher than in normal euvolemic controls (Cogan, 1984). The augmented HCO_3_^−^ reabsorption was attributed to increased activity of the sodium–hydrogen exchanger (NHE‐3; SLC9A3) and/or HCO_3_^−^ exit at the basolateral membrane (Cogan, 1984), presumably by the sodium/bicarbonate cotransporter (NBC‐e1; SLC4A4). The electroneutral sodium/bicarbonate cotransporter NBCn, expressed in the thick ascending segment of Henle's loop as well as at the basolateral membrane of *α*‐intercalated cells (Vorum et al., 2000), was upregulated by high CO_2_ when expressed in HEK cells (Ostrowski et al., 1993). In the collecting duct, an increase in the activity of H^+^‐ATPase was reported (Al‐Awqati, 1985) as well as a decrease in abundance of pendrin (Wagner et al., 2002; de Seigneux et al., 2007), a Cl^−^‐HCO_3_^−^ exchanger at the apical membrane of type‐B, and non‐A, non‐B intercalated cells. In the collecting duct, NH_4_^+^ is secreted by the NH_3_/NH_4_^+^ transporters, Rhbg and Rhcg, localized at the basolateral and apical membranes of the collecting duct cells, respectively (Nakhoul et al., 2003; Verlander et al., 2003; Nakhoul et al., 2005; Weiner and Verlander, 2014; Caner et al., 2015). The effect of respiratory acidosis on the Rh transporters in the distal nephron is not yet known.

In this study, we determined urinary excretion of NH_4_^+^ and TA in mice breathing high CO_2_ for 3 days. We also examined the effect of chronic hypercapnia on NH_3_/NH_4_^+^ transporters in the collecting duct. Our data indicate that prolonged hypercapnia has a significant effect on NH_4_^+^ excretion and that this effect is reflected in changes in expression of mechanisms involved in NH_3_/NH_4_^+^ and acid–base transport in the collecting duct.

## Methods

### Metabolic cages and in vivo studies

We conducted in vivo experiments on mice subjected to induced chronic respiratory acidosis. Two groups of mice, housed in metabolic cages, (five each, three replicates) were placed in special chambers (BioSpherix‐A) where breathing gas mixtures can be controlled. One group breathed 8% CO_2_ (21% O_2_ & 71% N2) to induce respiratory acidosis and the other breathed normal air as control (0.04% CO_2_, 21% O_2_, 79% N_2_).

Respiratory acidosis was confirmed by measurements of collected blood samples. Blood gases and ions were measured using i‐STAT blood analyzer. Urine samples were collected daily under oil. Urine samples were analyzed for pH, TA, and NH_4_^+^ measurements.

Urinary pH was measured using a glass pH combination microelectrode (MI‐415, microelectrodes Inc, Bedford, NH). Urinary TA and ammonium were measured using the method described by Chan (Chan, 1972) and briefly described here. To determine TA, 25 or 50 µL of urine samples was acidified by adding equal volume of 0.1N HCl and then titrating it back to pH 7.4 by adding 0.4N NaOH. TA was calculated as (volume of NaOH added/volume of sample) x 0.4N NaOH. The same protocol was applied in parallel to an equal volume of distilled water to correct for blank measurement. To measure urinary ammonium, 25 or 50 µL of urine was used to which an equal volume of 8% formaldehyde was added. Formaldehyde releases H^+^ from NH_4_^+^ as in this reaction: HCOH + NH_4_^+^ → NH(OH) NH_3_^+^ + H^+^. The sample is then titrated back to pH 7.4 with 0.1N NaOH to determine NAE as (Volume of NaOH/Volume of sample) x 0.1N NaOH. Total urinary NH_4_^+^ is then calculated as NAE‐TA and reported as total amount per day.

On day 4, mice were anesthetized by isoflurane and a terminal blood collection was obtained using cardiac puncture. Transcardial perfusion with phosphate buffer solution (PBS) cleared the kidneys from excess blood. The kidneys were harvested, placed in cold Ringer, and the capsule was removed. The kidneys were dissected to isolate cortex and medulla and the tissues were cut into several pieces and washed in ice‐cold PBS. All studies were approved by Tulane Institutional Animal Care and Use Committee.

### Western blots

For whole‐cell protein extraction, kidney tissues from cortex or medulla were lysed in Cell‐Lytic (Sigma) in the presence of protease inhibitors. All protein content was quantified using the Pierce BCA protein assay, and normalized to 1μg/μL. Proteins were separated using 10% sodium dodecyl sulfate‐polyacrylamide gel electrophoresis (SDS PAGE) under reducing conditions (Laemmli, 1970). Prestained molecular weight markers were run in parallel lanes (Li‐Cor, Lincoln, Nebraska). Experiments were run in duplicates or triplicates. After electrophoresis, proteins were transferred to a nitrocellulose membrane. The membranes were blocked for nonspecific binding, incubated overnight at 4°C with primary antibodies, washed, and incubated with secondary antibodies conjugated to a fluorescent dye (Li‐Cor). The primary antibodies used are listed below. Glyceraldehyde 3‐phosphate dehydrogenase (GAPDH) was used for normalization. For every gel, the normalized reading (ratio of protein of interest/ GAPDH) in tissues from control animals was set at one (100 %) and the normalized reading in the experimental condition was calculated as the ratio to one. The immunoreactive complex was visualized using Li‐Cor Odyssey Infrared System and analyzed by resident software. The membranes were stripped up to two times using Li‐Cor Stripping buffer as directed by the manufacturer, blocked and re‐probed as described above. Statistical analysis was performed using t‐test. The experiments were repeated at least three times on three different tissues.

### Antibodies

The Rhbg and Rhcg antibodies are a gift from Dr. David Weiner (University of Florida, Gainsville). The antibodies were raised in rabbit against a hydrophilic cytoplasmic region near the COOH terminus (Verlander et al., 2003). Carbonic anhydrase‐IV antibody is a rabbit polyclonal raised against amino acids 1–50 of CA‐IV (Santa Cruz Biotechnology, Inc). Pendrin antibody is a gift from Dr. Susan Wall (Emory University, Atlanta). This antibody was validated in tissues from pendrin knockout mice and successfully used in Western blots (Knauf et al., 2001; West et al., 2015).

### Immunohistochemistry

For frozen sections, kidney tissues were fixed by immersion in a periodate (10 mmol/L) lysine (70 mmol/L) paraformaldehyde (4%) (PLP) overnight (McLean and Nakane, 1974)). Tissues were then washed with PBS, incubated in 30% sucrose for 2 h, and mounted in O.C.T. compound (Sakura Finetek). Cryosections (5 µm) were rehydrated in PBS and pretreated with 1% SDS for 5 min in PBS to enhance the staining; they were then washed and blocked with serum. After incubation with the primary antibody, sections were incubated with the secondary antibody (Alexa fluor anti‐rabbit 488). Sections were then washed and counterstained with the nuclear marker 4’,6‐diamidino‐2‐phenylindole, dihydrochloride (DAPI), and mounted in Vectashield (Vector Laboratories, Burlingame, CA). For negative controls, sections were incubated without the primary antibody. Micrographs were obtained using a Nikon Eclipse 80i microscope and a Spot RT digital camera.

## Statistics

Values for Western blot analysis are reported as means ± SE of the mean. “n” is the number of replicate experiments. For every gel, the calculated densitometry values from control animals were averaged and set to one. Samples that came from different tissues were included in the total number of samples. Samples that came from the same tissue on the same gel were pooled together and averaged (duplicate samples). Experimental (hypercapnia) values, normalized to GAPDH, were calculated as a percent of control. Statistical significance was determined using two‐tailed unpaired Student's t‐test, unless mentioned otherwise. *P* < 0.05 was considered significant. Data of urinary excretion of NH_4_^+^ and TA were analyzed using ANOVA: two‐factor with replication.

## Results

In vivo experiments provided blood and urine measurements in two groups of mice (five each) placed in metabolic cages. As described in Methods, one group of mice breathed 8% CO_2_ for 3 days to induce chronic hypercapnia. The control group breathed normal air. Blood acid–base parameters from mice exposed to high CO_2_ (*n* = 15) had average values of pH, HCO_3_^−^, TCO_2_, and K^+^ of 7.16 ± 0.03; 22.2 ± 0.94 mmol/L; 24.2 ± 0.83 mmol/L; and 6.0 ± 0.48 mmol/L, respectively. In control mice breathing normal air, blood pH, HCO_3_^−^, TCO_2_, and K^+^ were 7.30 ± 0.04; 18.8 ± 1.9 mmol/L; 20.4 ± 2.0 mmol/L; and 4.4 ± 0.35 mmol/L, respectively. In hypercapnic mice, pH was lower, whereas TCO_2_ and K^+^ were significantly higher than in control mice (*P* < 0.05).

**Figure 1:**
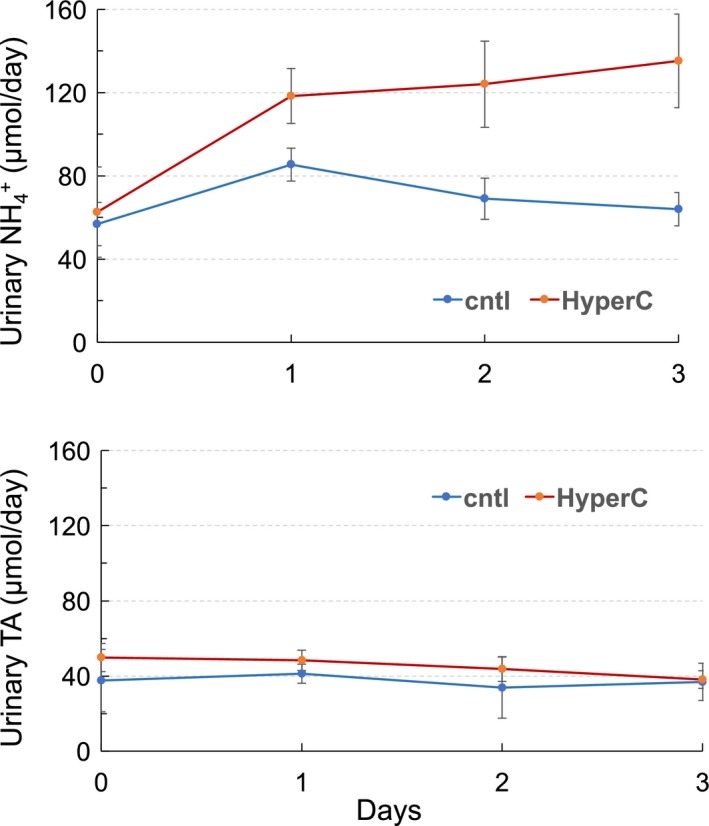
Urinary excretion of ammonium (NH_4_^+^) and titratable acids (TA) in response to hypercapnia**.** In mice breathing 8% CO_2_ (red tracing), NH_4_^+^ excretion increased significantly after 24 h and remained high in days 2 and 3 compared to control mice breathing normal air (blue tracing). The difference in urinary NH_4_^+^ excretion between control and hypercapnic animals over 3 days was highly significant (*P* < 0.01; ANOVA: two‐factor with replication). There was no significant change in TA excretion.

**Figure 2:**
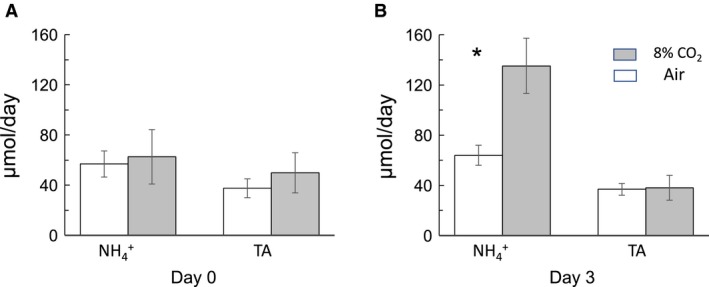
Summary bar graph of urinary excretion of NH_4_^+^ and TA in control (breathing air) and hypercapnic mice (breathing 8% CO_2_). (A) indicates that base‐line measurements (at day 0) of NH_4_^+^ excretion were similar in control (56.9 ± 10.4 µmol/day) and hypercapnic mice (62.6 ± 21.7 µmol/day). Excretion of TA was also similar in control (37.5 ± 7.5 µmol/day) and hypercapnic mice (49.8 ± 16.6 µmol/day); *n* = 15, *P* > 0.05. (B) on day 3, the amount of NH_4_^+^ excretion was 64.0 ± 8.0 µmol/day in mice breathing air but increased significantly to 135.2 ± 22.5 µmol/day in mice breathing 8% CO_2_ (*n* = 15, *P* < 0.05). There was no significant difference in TA excretion between control mice (36.9 ± 4.7 µmol/d) and mice breathing 8% CO_2_ (38.1 ± 9.9 µmol/d); *n* = 15, *P* > 0.05. * indicates *P* < 0.05 of hypercapnic group compared to the group breathing air.

Urinary excretion of NH_4_^+^ and TA were determined daily. As shown in Figure 1, urinary NH_4_^+^ in hypercapnic mice increased significantly after 24 h and remained high in days 2 and 3. In control mice, there was a small increase in urinary NH_4_^+^ excretion in day 1 (*P* < 0.05) but leveled off in days 2 and 3. There was no significant changes in urinary excretion of TA in both hypercapnic or control groups. Urinary excretion of NH_4_^+^ in hypercapnic mice was significantly higher than in control mice (*P* < 0.01, ANOVA: two‐factor with replication). Figure 2 is a summary bar graph showing urinary NH_4_^+^ and TA excretion at days 0 and 3 in both groups of mice.

### Effect of hypercapnia on Rhbg and Rhcg expression

**Figure 3:**
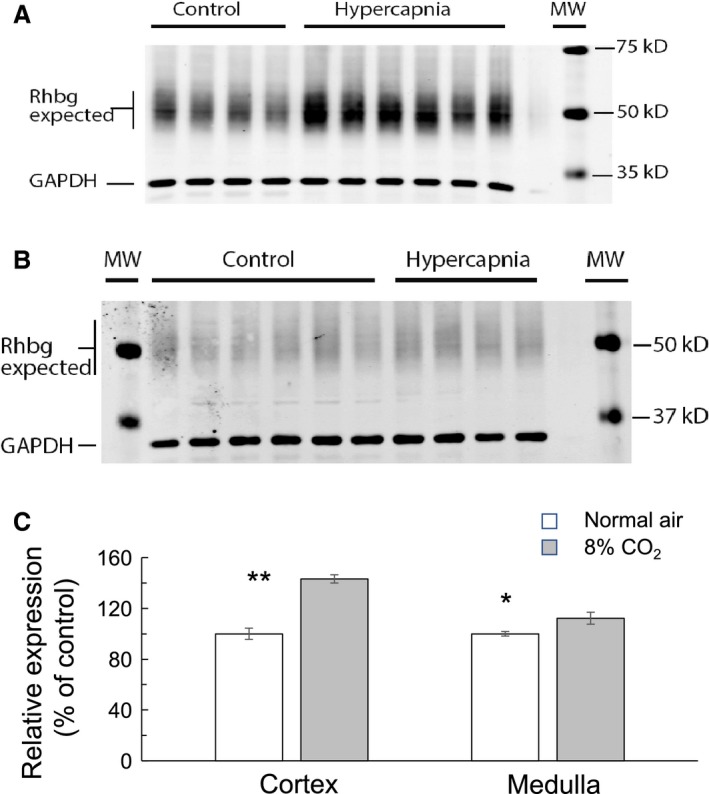
Semiquantitative immunoblots of Rhbg expression in kidneys from mice breathing air or during chronic hypercapnia (8% CO_2_). (A) Western blots from renal cortex show that Rhbg protein expression in control mice (lanes 1–4) was less than that in hypercapnic mice (lanes 5–10). (B) Rhbg expression in renal medulla was also less in control mice (lanes 2–7) compared to hypercapnic mice (lanes 8–11). Lanes 1 and 13 are molecular weight markers. (C) Graphical representation of Rhbg protein expression as a ratio of Rhbg to GAPDH indicating an increase in Rhbg protein expression by 44% ± 3% in cortex (*n* = 10, *P* < 0.001) and by 12% ± 4% in medulla (*n* = 10, *P* < 0.05) in mice breathing high CO_2_. ** indicates *P* < 0.001 and * indicates *P* < 0.05.

We performed Western blot analysis to determine the effect of hypercapnia on protein abundance of Rhbg. The abundance of Rhbg protein was determined separately in isolated kidney cortex and medulla. Figure 3A shows that hypercapnia induced a marked increase in the expression of Rhbg in renal cortex compared to control. Similarly, protein abundance of Rhbg in renal medulla also increased (Fig. 3B). Figure 3C is a bar graph indicating that hypercapnia caused Rhbg protein abundance to increase from 100 ± 4% to 144 ± 3% in cortex (*n* = 10, *P* < 0.001) and to 112 ± 4% in medulla (*n* = 10, *P* < 0.05).

**Figure 4:**
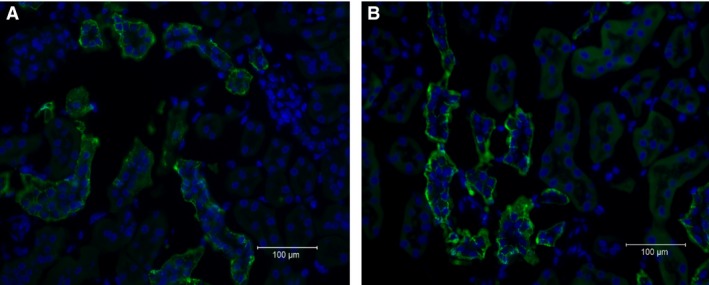
Immunolabeling of Rhbg in kidneys of mice breathing air (A) or 8% CO_2_ (B). The labeling of Rhbg in the renal cortex (green fluorescence) was observed in the basolateral plasma membrane of intercalated cells of the cortical collecting duct (CCD), as expected, and was more intense in kidney sections from hypercapnic mice (B) as compared to sections from mice breathing air (A). Magnification was 400X.

To further confirm the effect of hypercapnia, we labeled Rhbg in kidney slices obtained from control mice (breathing air) and hypercapnic mice (breathing 8% CO_2_). As shown in Figure 4, Rhbg staining (green) at the basolateral membrane was more intense in sections from hypercapnic mice (Fig. 4B) as compared to control (Fig 4A).

**Figure 5:**
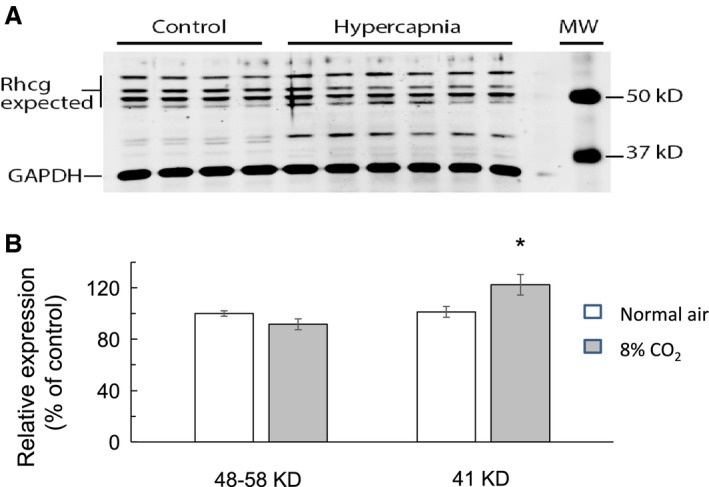
Western blots of Rhcg protein expression in renal cortex. Rhcg blots typically showed multiple bands between 48–58 KD (expected), however, there was also another band at 41 KD. (A) Rhcg protein expression at 48–58 KD in control (lanes 1–4) was not significantly different compared to hypercapnia (lanes 5–10). However, the band intensity at 41 KD was less in control mice (lanes 1–4) as compared to hypercapnic mice (lanes 5–10). (B) Summary graph of Rhcg protein expression as a ratio of Rhcg to GAPDH. In hypercapnic mice, Rhcg expression (at 48–58 KD) decreased slightly from 100.0 ± 0.2% to 90.1 ± 5.3 % in control but the change was not statistically different (*P* > 0.05, *n* = 10). However, at 41 KD there was an increase in expression by 22.4 ± 6.6% in hypercapnia as compared to control mice (*n* = 10, *P* < 0.01). * indicates significance at *P* < 0.01.

Rhcg expression in response to hypercapnia showed a different response between cortex and medulla and a unique pattern of bands by Western blot. Figure 5A is an immunoblot showing protein abundance in cortex of control mice (lanes 1–4) and in hypercapnic mice (lanes 5–10). The antibodies against Rhcg produced several bands; specific bands of 48–58 KD and another band at 41 KD. The bands at 48–58 KD were quantified together. The intensity of the bands at 48–58 KD was slightly decreased in hypercapnia (lanes 5–10) but the change was not statistically significant. However, the band at 41 KD showed a significant increase in hypercapnia compared to control. Figure 5B indicates relative changes in the expression of Rhcg in the renal cortex in response to chronic hypercapnia from 100.0 ± 0.2 % to 90.1 ± 5.3 % at 48–58 KD (*n* = 10, *P*> 0.05, N.S.) and to 122.4 ± 6.6 % (*n* = 10, *P* < 0.01) at 41 KD.

**Figure 6:**
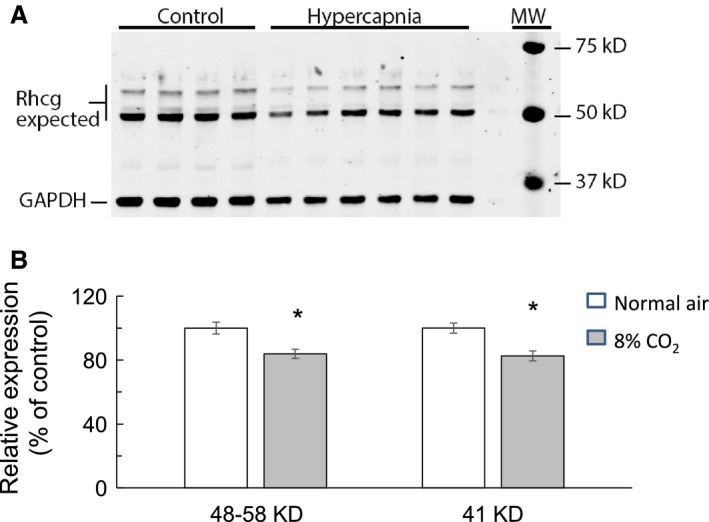
Western blot of Rhcg protein expression in renal medulla. (A) Rhcg protein expression, calculated from 48–58 KD bands, was higher in renal medulla of control mice (lanes 1–4) compared to mice breathing 8% CO_2_ (lanes 5–10). There was a faint band at 41 KD. (B) Summary bar graph showing that Rhcg protein expression in renal medulla of hypercapnic mice was decreased by 16.1 ± 2.9% (at 48–58 KD) and by 17.4 ± 3.1% (at 41 KD) as compared to control mice. *n* = 10, *P* < 0.005. * indicates significance at *P* < 0.005.

Figure 6A shows Rhcg abundance in medullary tissue of control mice (lanes 1–4) and in mice breathing high CO_2_ (lanes 5–10). Rhcg expression in the medulla was significantly decreased in hypercapnia as compared to control. Figure 6B shows that relative expression of Rhcg decreased from 100.0 ± 3.7 % to 83.9 ± 2.9 % at 48–58 KD and to 82.6 ± 3.1 KD at 41 KD (*n* = 10, *P* < 0.005).

### Effect of hypercapnia on CA‐IV expression

**Figure 7:**
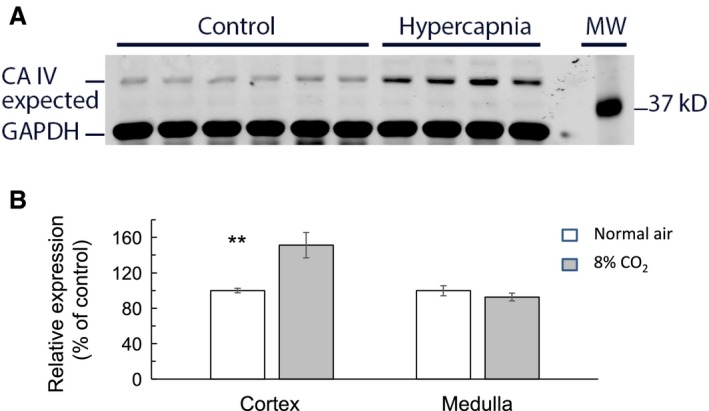
Effect of hypercapnia on renal expression of carbonic anhydrase IV (CA‐IV) by Western blot analysis. (A) Typical western blot showing that hypercapnia caused an increase in CA‐IV expression in renal cortex (lanes 7–10) compared to control (lanes 1–6). Lane 12 shows molecular weight standards. (B) Summary graphs indicating that relative protein expression of CA‐IV in mice breathing 8% CO_2_ increased by 51.3 ± 14.3% in renal cortex (*n* = 10, *P* < 0.005) compared to control mice breathing air. There was no significant change in relative protein expression of CA‐IV in renal medulla. ** indicates *P* < 0.005.

CA‐IV is the membrane‐bound isoform of carbonic anhydrase that has been reported to play a significant role in facilitating H^+^ secretion in the kidney. CA‐IV is expressed in both cortex (mainly in proximal tubules and to a lesser extent in collecting duct) and medulla (collecting duct). We performed Western blot analysis on cortex and medulla to determine relative abundance of CA‐IV in response to hypercapnia. As shown in Figure 7A, an immunoblot labeled for CA‐IV showed a weak band at 40 KD (expected band) in control cortex (lanes 1–6) that readily increased in hypercapnia (lanes 7–10).

In tissues of the renal medulla, there was no significant change in the band intensity at 40 KD due to hypercapnia. These data are summarized in Figure 7B and indicate that chronic hypercapnia increased the relative expression of CA‐IV only in cortex from 100.0 ± 2.6% to 151.3 ± 14.3% (*n* = 10, *P* < 0.005) but not in medulla.

### Effect of hypercapnia on Pendrin

**Figure 8:**
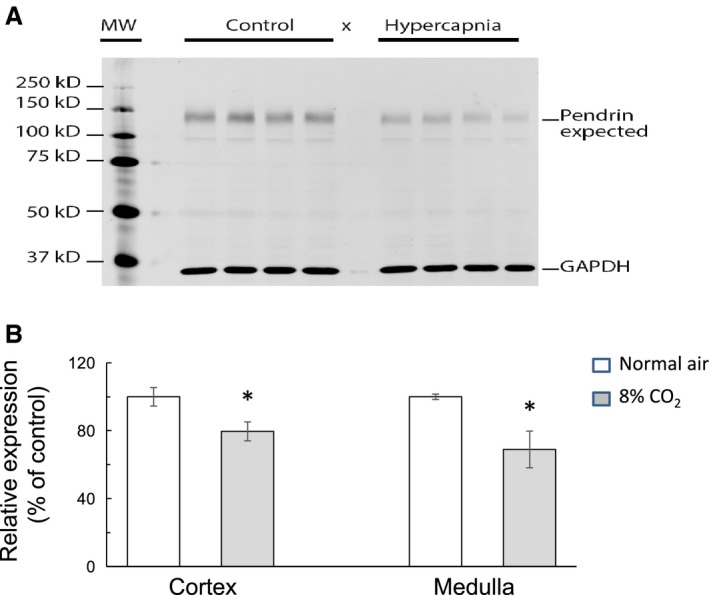
Effect of hypercapnia on renal expression of pendrin. (A) Western blot on tissue lysates of renal medulla showing bands specific for pendrin at ~130 KD from control (lanes 3–6) and hypercapnic mice (lanes 8–11). (B) Summary graph of relative pendrin band density as a ratio of pendrin to GAPDH indicating that hypercapnia caused a significant decrease in expression of pendrin in cortex (by 20.4 ± 4.5%) and medulla (by 30.6 ± 7.1%) compared to control (*P* < 0.05). Tissue lysates were obtained from four hypercapnic and six control mice. * indicates *P* < 0.05.

Pendrin, a Cl^−^‐HCO_3_^−^ exchanger, is located at the apical membrane of type B and nonA – nonB intercalated cells. Pendrin is thought to facilitate HCO_3_^−^ secretion and to contribute to acid–base regulation. In our study, we investigated whether chronic hypercapnia affected the abundance and expression of renal pendrin. As shown in Figure 8A, hypercapnia for 3 days decreased the expression of pendrin in the renal medulla (lanes 8–11) compared to control (lanes 3–6). Chronic hypercapnia also reduced pendrin expression in renal cortex. These data are summarized in Figure 8B indicating a decrease in relative abundance of pendrin in cortex from 100 ± 5.5% to 79.6 ± 5.6% (*P* < 0.05) and from 100 ± 1.6% to 69.0 ± 10.8% (*P* < 0.05) in medulla.

## Discussion

Kidney plays a major role in excreting acid to correct for systemic acid loads. The cornerstone of this role is the capacity of the kidney to produce and excrete total ammonia (NH_3_/NH_4_^+^). Excretion of NH_3_/NH_4_^+^ is ultimately determined by changes in renal ammonia production (mainly in the proximal tubule) and potentially a change in net NH_3_/NH_4_^+^ transport (along the nephron and mainly in the collecting duct). Our study focused on the latter. We demonstrated that chronic hypercapnia increased the net urinary ammonium excretion, upregulated the expression of Rhbg, and modified the expression of Rhcg, two novel NH_3_/NH_4_^+^ transporters expressed in the collecting duct. This study is among the first to demonstrate an effect of chronic high CO_2_ on specific NH_3_/NH_4_^+^ transporters in the kidney.

Renal ammonia production in response to acid–base changes has been extensively studied in various species, including humans. The general consensus, however, is that, ammoniagenesis and glutamine utilization (the source for ammonia production) are increased in response to acute metabolic and respiratory acidosis (Vinay et al., 1980; Narins et al., 1982). The data suggest that a decrease in intracellular pH (pH_i_) of the proximal tubule is the main cause for the increase in ammoniagenesis (Trivedi and Tannen, 1986).

Ammoniagenesis, in response to chronic acidosis, is more complicated due to predominant adaptive changes in metabolic pathways involved in ammonia production. However, it is firmly established that chronic metabolic acidosis increases renal capacity to produce ammonia. This has been readily demonstrated in the rat, dog, and human both in vivo and in vitro studies (Tannen, 2011). Although low pH may play a direct role, other factors including increased activity of enzymes involved in glutamine deamination and metabolism such as phosphoenolpyruvate carboxykinase (PEPCK) and phosphate‐dependent glutaminase (PDG) have been demonstrated (Tannen, 1978). An important difference is observed when comparing chronic metabolic and respiratory acidosis. In chronic respiratory acidosis, the rate of urinary ammonium excretion, while less than in chronic metabolic acidosis, is reported to be increased in dog and rat (Carter et al., 1959; Polak et al., 1961; Schwartz, 1965; Rodriguez‐Nichols et al., 1984) but less certain in mouse or human. Since intracellular pH supposedly recovers in chronic respiratory acidosis compared to chronic metabolic acidosis, the divergent response in ammoniagenesis was attributed to the difference in pH_i_ of the proximal tubule. The effect of chronic acidosis, metabolic or respiratory, on NH_3_/NH_4_^+^ transport, rather than production, is less clear.

It is well accepted that mechanisms that lower urine pH will result in increased ammonium excretion even when ammoniagenesis by the kidney is unchanged. In chronic hypercapnia studies, there was an increase in the capacity of the distal nephron to secrete H^+^ (Batlle et al., 1985; Tannen and Hamid, 1985). Increased H^+^ secretion was demonstrated in the proximal tubule also, as would be expected for the need to increase HCO_3_^−^ reabsorption in response to chronic respiratory acidosis (Sullivan and Dorman, 1955; Ullrich and Papavassiliou, 1981).

Chronic hypercapnia induces chronic acidosis in neonatal and adult mammals (Caso et al., 2005; Kantores et al., 2006). Although altered changes in metabolism and ammoniagenesis may contribute to renal adjustment to respiratory acidosis, the major factor in the compensation is the activity and level of expression of plasma membrane acid transporters. Changes in expression of transporters (NH_3_/NH_4_^+^ mechanisms in this case), rather than metabolism (e.g. ammoniagenesis), are likely to become more important with prolonged hypercapnia (Boron, 2004; Kanaan et al., 2007). In our study, we focused on the response of the NH_3_/NH_4_^+^ transporters (Rhbg and Rhcg) in the collecting duct.

### Rhbg

The role of Rhbg in renal NH_3_/NH_4_^+^ transport is firmly established. However, several mechanisms have been proposed to explain function. Some studies report that NH_3_/NH_4_^+^ transport by Rhbg is electroneutral and behaves as an NH_4_^+^–H^+^ exchanger (Ludewig, 2004; Zidi‐Yahiaoui et al., 2005; Mak et al., 2006). Our studies demonstrated that Rhbg transports NH_3_ and NH_4_^+^ independently and that this transport is electrogenic (Nakhoul and Hamm, 2004; Nakhoul et al., 2006; Nakhoul et al., 2010).

The effect of acid–base disturbances on NH_3_/NH_4_^+^ transport by Rhbg has shown mixed results. In the mouse, one study reported that metabolic acidosis increased mRNA of Rhbg but the effect on Rhbg protein expression was not known (Cheval et al., 2006). Increased Rhbg mRNA was demonstrated in acid‐loaded mice (by HCl‐fortified diet) but only in mice with genetic deletion of Rhcg (Lee et al., 2009; Lee et al., 2010). In another study, genetic deletion of pendrin (expected to cause alkalosis) decreased Rhbg expression (Kim et al., 2005), consistent with the need to decrease acid excretion.

The effect of respiratory acid–base disturbances on Rhbg and NH_3_/NH_4_^+^ transport has not been reported. Our data show that prolonged hypercapnia increased the net ammonium excretion coupled to increased expression of Rhbg in cortex and medulla. This is consistent with a role of Rhbg in contributing to enhanced NH_3_/NH_4_^+^ excretion under these conditions.

The significant effect of high CO_2_ on the expression of Rhbg raises another possibility. Few studies suggested that some Rh analogues (Rh1, Amt) may transport CO_2_ (Kaplan et al., 2004; Soupene et al., 2004; Geyer et al., 2013). Our data showed that CO_2_ caused a faster and bigger pH_i_ decrease in oocytes expressing Rhbg as compared to H_2_O‐injected oocytes (Nakhoul et al., 2017) suggesting that Rhbg facilitates CO_2_ transport. If Rhbg is a CO_2_ transporter, this raises the possibility that elevated CO_2_ (in chronic hypercapnia) may by itself regulate the expression of Rhbg. However, one study in rainbow trout showed that high CO_2_ did not directly elicit changes in mRNA transcription levels of Rhbg2 (the Rh protein identified in fish) in the gill or skin (Nawata and Wood, 2008). On the other hand, there was an increase in Rhbg2 mRNA in response to high ammonia in plasma (Nawata et al., 2007). No other studies on mammalian tissues and differential effects of high CO_2_ or NH_3_ have been reported.

### Rhcg

In our study, the effect of chronic respiratory acidosis on Rhcg expression showed an unexpected pattern. High CO_2_ did not cause a statistically significant change in Rhcg expression in the renal cortex (at the expected bands of 48–58 KD), whereas expression in the medulla was significantly reduced. However, there was an increase in band intensity at 41 KD. Whether this band represents a shift in glycosylation of Rhcg in response to high CO_2_ is not yet known. Several studies indicate that Rhcg expression generally parallels NH_3_/NH_4_^+^ excretion. However, renal cellular expression of Rhcg has been a confounding factor that sometimes complicated the metabolic results. Unlike Rhbg, that is predominately expressed at the basolateral membrane in *α*‐intercalated cells, Rhcg is expressed in different cell types and at different cellular locations. In human, rat, and mouse kidneys, Rhcg has been located at both apical and basolateral membranes and even in subapical vesicles (Han et al., 2006; Seshadri et al., 2006a; Brown et al., 2009; Kim et al., 2009). Rhcg is expressed in all segments of the distal tubule and collecting duct (Eladari et al., 2002; Verlander et al., 2003) in both intercalated cells and principal cells.

This unusual pattern of Rhcg localization often resulted in nonuniform expression at the membrane (apical vs. basolateral) or cell (intercalated vs. principal cells) or segment (medullary vs. cortical) of the nephron. In mouse studies, these differences in expression seem to correlate with differing ability to excrete NH_4_^+^ in response to acid loads (Weiner and Verlander, 2010). For example, chronic metabolic acidosis significantly increased the Rhcg protein expression but not mRNA, in outer medullary collecting duct (OMCD) and inner medullary collecting duct (IMCD) but not in the cortex (Seshadri et al., 2006a; Seshadri et al., 2006b). Other studies reported increased basolateral membrane expression of Rhcg in chronic metabolic acidosis and hypokalemia (Kim et al., 2007; Han et al., 2011). However, the process of increased Rhcg expression differed among cell types. For example, in OMCD‐intercalated cells, a change in subcellular distribution of Rhcg was evident, whereas increased protein expression was predominant in OMCD principal cells.

It is likely that similar patterns of varied response in expression of Rhcg occur in response to chronic respiratory acidosis. It is not clear what the mechanisms that induce such differential responses in expression are. One possibility is that the varied CO_2_ gradient along the cortical–medullary axis could lead to different changes in intracellular pH in medullary and cortical cells that may explain the differences in Rhcg expression. This possibility needs to be examined further.

### CA‐IV and pendrin

Our data also showed that hypercapnia significantly increased the CA‐IV expression in the cortex but the effect on medullary expression was not statistically significant. CA‐IV is the membrane‐bound isoform of carbonic anhydrase and is abundantly expressed in the apical and basolateral membranes of the proximal tubule where it has an important role in facilitating HCO_3_^−^ reabsorption. CA‐IV expression was also demonstrated in the apical membrane of α‐intercalated cells of the cortical collecting duct (CCD), OMCD, and IMCD of rabbit (Schwartz et al., 2000) and in CCD and OMCD of the human kidney (Lonnerholm and Wistrand, 1991). The role of CA‐IV in the collecting duct is not well understood but it is thought to facilitate H^+^ secretion. As such, it may have a role in promoting NH_3_/NH_4_^+^ secretion in the collecting duct, although physiologic regulation of CA‐IV expression is not thoroughly examined. Some studies showed that metabolic acidosis increased the expression of mRNA of CA‐IV (Winkler et al., 1997; Tsuruoka et al., 1998). However, it is thought that the main effect is in the proximal tubule where CA‐IV would contribute to HCO_3_^−^ reabsorption in this case (Schwartz, 2002).

In one interpretation, the absence of apical CA‐IV (which catalyzes the dissociation of H_2_CO_3_ into CO_2_ and H_2_O) causes H^+^ concentration in the luminal fluid to increase above equilibrium due to slow dissipation of secreted H^+^ into CO_2_ and H_2_O (H^+^ + HCO_3_^−^ → H_2_CO_3_ → CO_2_ + H_2_O). The increased luminal H^+^ concentration, causing disequilibrium pH (Star et al., 1987a; Star et al., 1987b), can facilitate conversion of secreted NH_3_ to NH_4_^+^ and preventing the buildup of a lumen to cell NH_3_ gradient. However, this does not favor reabsorption of HCO_3_^−^. Alternatively, a more plausible possibility is that in chronic hypercapnia, the generation of H^+^ from the high CO_2_ is facilitated by apical CA‐IV. This will enhance conversion of NH_3_ to NH_4_^+^ and provides for a favorable gradient for NH_3_ secretion. This is consistent with our data indicating an increase in CA‐IV in chronic hypercapnia. Since our data demonstrated a significant increase in CA‐IV expression only in the cortex, it remains possible that the predominant effect of hypercapnia in this case is on proximal tubule rather than on collecting duct.

Pendrin (SLC26A4) is a Cl^−^‐HCO_3_^−^ exchanger located in the apical membrane of type B (ß‐IC) and nonA ‐ nonB intercalated cells of the collecting duct (Kim et al., 2005). In one study, metabolic acidosis induced by oral NH_4_Cl intake caused downregulation of pendrin, whereas oral HCO_3_^−^ loading caused an increase in pendrin‐positive cells in the mouse kidney (Wagner et al., 2002). Another study showed that hypoxic hypercapnia also caused downregulation of pendrin in CCD and CT (de Seigneux et al., 2007). Our study showed that prolonged hypercapnia at normal O_2_ decreased the abundance of pendrin in cortex and medulla. These data indicate that high CO_2_ acidosis downregulates pendrin even though plasma HCO_3_^−^ is high. This suggests that regulation of pendrin is probably directly regulated by acid–base imbalance and not by HCO_3_^−^ load. The downregulation of pendrin in this case may actually contribute to maintaining compensatory high plasma HCO_3_^−^ in chronic respiratory acidosis.

In summary, we have demonstrated that renal adaptation to chronic hypercapnia caused a net increase in ammonium excretion and altered the expression of membrane proteins that are critical for acid–base transport in the collecting duct. There was a decrease in abundance of the anion exchanger pendrin consistent with expected downregulation of an acid‐loading mechanism. The effect of hypercapnia on CA‐IV expression, an increase in cortex but not in the medulla, was unexpected. Whether this differential effect is cell‐specific or varied among different nephron segments is unclear and need to be investigated further. Importantly, we showed that hypercapnia caused a varied pattern of Rhcg expression in cortex and medulla but induced a significant increase in the expression of the NH_3_/NH_4_^+^ transporter Rhbg. This indicates that adaptation to chronic hypercapnia involves upregulation of NH_3_/NH_4_^+^ transport in the collecting duct and not merely an increase of ammoniagenesis in the proximal tubule.

## Conflict of Interest

The authors declare that they have no conflict of interest with the contents of this article.
